# Targeted therapy for head and neck squamous cell carcinoma microenvironment

**DOI:** 10.3389/fmed.2023.1257898

**Published:** 2023-08-30

**Authors:** Zhaomeng Guo, Kang Li, Peng Liu, Xiangmin Zhang, Jie Lv, Xianhai Zeng, Peng Zhang

**Affiliations:** ^1^Department of Otorhinolaryngology, Longgang Otorhinolaryngology Hospital and Shenzhen Key Laboratory of Otorhinolaryngology, Shenzhen Institute of Otorhinolaryngology, Shenzhen, Guangdong, China; ^2^Department of Graduate and Scientific Research, Zunyi Medical University Zhuhai Campus, Zhuhai, Guangdong, China; ^3^School of Computer Science and Engineering, Yulin Normal University, Yulin, Guangxi, China

**Keywords:** HNSCC, microenvironment, targeted therapy, tumor progression, inflammation

## Abstract

Head and neck squamous cell carcinoma (HNSCC) originates from the squamous epithelium of the oral cavity, oropharynx, larynx, and hypopharynx. HNSCC in the oral cavity and larynx is strongly associated with tobacco smoking and alcohol consumption, while oropharyngeal cancer is increasingly attributed to infection by human papillomavirus (HPV), particularly HPV-16. The tumor microenvironment (TME) is a complex network of cancer cells, immune cells, stromal cells, surrounding blood vessels, and signaling molecules, and plays a critical role in tumor cell survival, invasion, and recurrence. Therefore, it is critical to elucidate the molecular basis of the interaction between tumor cells and the TME in order to develop innovative anti-cancer therapeutic strategies.

## 1. Introduction

Head and neck cancer ranks as the sixth most common cancer globally, with approximately 600,000 new cases diagnosed every year. Head and neck squamous cell carcinoma (HNSCC) is the predominant type, and arises from the mucosal epithelium of the oral cavity, pharynx, and larynx (1, 2). Several risk factors of HNSCC have been identified, such as exposure to tobacco-derived carcinogens and excessive alcohol consumption (3). In addition, oncogenic viruses such as high-risk human papillomavirus (HPV), particularly HPV-16, are increasingly being recognized as common causes of HNSCC in younger patients (4). The treatment options for HNSCC include surgery, radiation therapy, chemotherapy, targeted therapy, or a combination thereof, and the suitable approach depends on the tumor location and staging, along with the age and overall health of patients (5). Nevertheless, the prognosis for HNSCC patients is often poor due to high rates of local recurrence and lymph node metastasis (6). The five-year survival rate of HNSCC patients ranges from 50 to 60%, and up to 30% will experience cancer recurrence and treatment failure (7).

**Figure 1:**
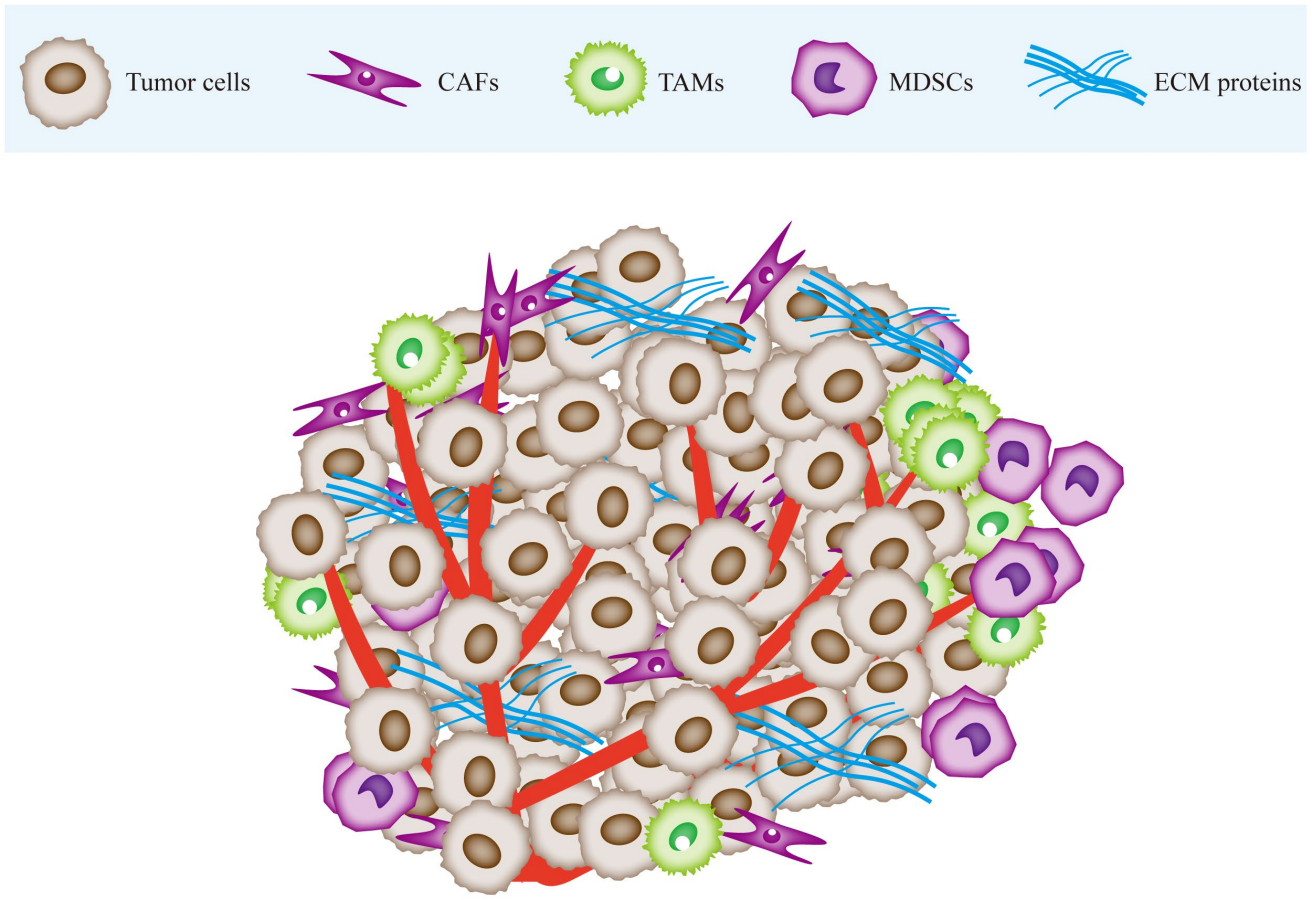
The tumor microenvironment is mainly composed of cancer cells, immune cells, stromal cells and extracellular matrix (ECM). Tumor tissue infiltrated by CAF and immune cells. Tumor tissue is stiffer than normal tissue due to stromal deposition and cross-linking. CAF, cancer-associated fibroblasts; TAM, tumor-associated macrophages; MDSC, myeloid-derived suppressor cells; ECM, extracellular matrix.

The tumor microenvironment (TME) is a complex array of cellular and non-cellular components that drive tumor initiation and progression (8). The cellular components include stromal cells and immune cells, and the non-cellular components consist of extracellular matrix (ECM) proteins (9). Stromal cells include cancer-associated fibroblasts (CAFs), endothelial cells (ECs), and the blood and lymphatic vessel network, while immune cells comprise of tumor-associated macrophages (TAMs), myeloid-derived suppressor cells (MDSCs), T cells, B cells, and natural killer (NK) cells. Tumor cells rely on the TME for nutrients, intermediate metabolites, hormones, cytokines/chemokines, and growth factors crucial for their proliferation and survival. Moreover, the TME plays a pivotal role in tumor immune evasion and promoting tumor-associated inflammation (10). On the other hand, the metabolic alterations in the proliferating tumor cells can reshape the TME to create conditions favorable for tumor progression (11) (Figure 1).

While surgery and radiation therapy are effective against early-stage tumors (stages I and II), many HNSCC patients are diagnosed at the advanced stage of the disease without a clinical history of precancerous lesions, which portends poor prognosis (12). Furthermore, Radiotherapy and chemotherapy often leads to severe side effects and reduces quality of life (13). Targeted therapy involves specific drugs that selectively bind to oncogenic targets within tumor cells, with minimal effects on the adjacent healthy tissues (14). Given the indispensable role of the TME in tumor progression, further research has been initiated into new therapeutic strategies that target TME for the treatment of HNSCC or other solid tumors.

## 2. Targeting the tumor microenvironment

Tumor microenvironment is a complex network of cellular and non-cellular components. Cancer is considered to be an evolutionary and ecological process involving continuous, dynamic and reciprocal interactions between cancer cells and TMEs (15). The TME is a key determinant of cancer prognosis and treatment outcomes (16). While the TME promotes tumor progression, the latter induces adaptations in the TME to facilitate its growth. These reciprocal interactions between tumor cells and the TME collectively shape the trajectory of the tumor (17). Furthermore, the TME becomes highly complex and heterogenous in the advanced stages of solid tumors (18). Therefore, it is crucial to elucidate the molecular interactions between tumor cells and the TME in order to identify potential therapeutic targets for cancer treatment. The key components of the TME that contribute to tumor progression, as well as the clinical studies on drugs targeting these components, have been discussed in the subsequent sections.

### 2.1. Targeting the extracellular matrix

The ECM is an intricate network of protein, polysaccharides, and glycoproteins that provides structural and biochemical support to the tissue. It is primarily composed of collagen, along with fibronectin, elastin, laminin, hyaluronic acid, chondroitin sulfate, keratan sulfate, and heparan sulfate (19). Dysregulation of the ECM is a hallmark feature of cancer (20). Tumor cells recruit and transform fibroblasts into CAFs, which contribute to excessive ECM deposition. CAFs are the predominant non-immune cells in the TME, and constitute up to 80% of the cells in advanced HNSCC tumors. While undifferentiated fibroblasts can suppress tumor growth, activated CAFs remodel the tumor stroma, and influence the behavior and invasiveness of HNSCC cells by producing soluble factors and ECM proteins (21, 22). Excessive collagen deposition and crosslinking of fibrillar collagen and elastin result in a dense and rigid ECM, leading to tissue stiffening (23). This protein network protects tumor cells from immune destruction and mediates treatment resistance. Moreover, the ECM promotes tumor progression by providing proliferative signals to the tumor cells, blocking growth-inhibitory factors, inducing angiogenesis, and facilitating the invasion and metastasis of tumor cells (24).

Given its critical role in HNSCC progression, the ECM represents an important therapeutic target. The TGF-β signaling pathway is involved in collagen synthesis (25), and drugs targeting TGF-β receptors have shown promising clinical effects. Fluorothiazinone (FT), a plant-derived anti-bacterial alkaloid, can inhibit collagen synthesis by inactivating the TGF-β/Smad2/3 signaling pathway (26). Wang et al. demonstrated that HF inhibited the proliferation of CAFs in oral squamous cell carcinoma (OSCC) by targeting the TGF-β/Smad2/3 pathway (27). Flumatinib (HF) has shown favorable clinical outcomes. Bintrafusp alfa, a bifunctional fusion protein targeting TGF-β and PD-L1, achieved promising clinical outcomes in a phase I trial in advanced HNSCC patients with a manageable safety profile. Darantelcept binds to activin receptor-like kinase 1 (ALK1), a TGF-β receptor expressed on activated endothelial cells, and blocks TGF-β signaling. It has demonstrated modest dose-dependent anti-cancer activity and a favorable safety profile in phase I clinical trials in patients with cisplatin-resistant, recurrent or metastatic HNSCC (RM-HNSCC), and may be tested further in combination with radiotherapy in RM-HNSCC patients (28).

CD44 is a receptor for hyaluronic acid, collagen, fibronectin and growth factors, and thus regulates signaling pathways related to cancer proliferation, invasion, metastasis, and treatment resistance (29). CD44 isoforms are overexpressed in various tumors, including HNSCC. Although targeted drugs like bivatuzumab mertansine (BIWI 1) have been explored in clinical trials, their severe skin toxic side effects have halted their development (30). The strong toxic side effects of BIWI 1 have forced the termination of research on this drug. However, CD44 plays an important role in tumor progression and has the potential to be a tumor therapeutic target, which may warrant more in-depth research in tumor therapy in the future.

In summary, dysregulation of the ECM contributes to cancer development and progression, and targeting the ECM and associated signaling pathways is a promising therapeutic strategy for HNSCC. However, further research and clinical studies are necessary to unravel the intricate interplay between tumor cells and the ECM in order to develop effective and safe targeted therapies against HNSCC.

### 2.2. Targeting tumor hypoxia

Tumor hypoxia (TH) is characterized by an increased demand for oxygen due to the rapid proliferation of tumor cells and is often associated with poor prognosis (31). It can be classified into acute and chronic hypoxia (32). Acute hypoxia is the result of insufficient oxygen supply to cells due to compromised blood vessels, while chronic hypoxia is primarily caused by limited oxygen diffusion into the tumor cells on account of the distance from blood vessels or restrictive geometric shapes (33). Chronic hypoxia is more common in solid tumors due to their expansive growth. For instance, the oxygen pressure within HNSCC tissue is <10 mm Hg compared to approximately 43 mm Hg in normal tissues (34).

Hypoxia exacerbates the malignant phenotype of tumor cells and inhibits apoptosis, thereby promoting tumor progression, invasion, metastasis, and treatment resistance (35, 36). Moreover, hypoxia-induced increase in glycolysis and carbon dioxide production acidifies the TME, which renders cells resistant to radiation and chemotherapy (37). Key endogenous hypoxia markers in tumors include hypoxia-inducible factor 1 (HIF-1), glucose transporter 1 (GLUT-1), carbonic anhydrase IX (CAIX), vascular endothelial growth factor (VEGF), and osteopontin (OPN) (38). HIF-1 is a heterodimeric transcription factor composed of a constitutively expressed β subunit and an oxygen-regulated α subunit. It is a major regulator of cellular oxygen homeostasis, and promotes angiogenesis in hypoxic tumor tissues by upregulating VEGF and promoting recruitment of mature endothelial cells (39). HIF-1 also induces glycolysis and GLUT-1 expression under hypoxic conditions to facilitate energy production (40, 41). OPN expression is induced under hypoxic conditions independent of the HIF pathway, and protects cells against hypoxia-triggered death (42). CAIX is a cell surface metalloenzyme that catalyzes the reversible conversion of carbon dioxide to bicarbonate (HCO3-) and H+, which maintains a favorable pH for tumor cell survival and growth. Furthermore, CAIX contributes to extracellular acidification, and promotes tumor cell migration, invasion, metastasis, and treatment resistance (43).

Hypoxia and TME acidification are contributing factors to HNSCC recurrence (44). Furthermore, hypoxic conditions promote epithelial-mesenchymal transition (EMT) of OSCC cells, leading to a significant decrease in E-cadherin mRNA levels and increased tumor cell migration (45). Clinical trials targeting HIF-1 and CAIX have been conducted extensively and have yielded some promising therapeutic results. Thus, HIF-1 and CAIX might be promising therapeutic targets for head and neck cancers. For instance, the HIF-1α inhibitor bortezomib has shown good tolerability in combination with bevacizumab in phase I trials for advanced refractory malignancies. It is effective against pre-treated advanced malignancies and inhibits tumor angiogenesis. Furthermore, clinical trials involving bortezomib in combination with docetaxel for androgen-independent prostate cancer and the combination of bortezomib and irinotecan for relapsed/refractory high-risk neuroblastoma have reported encouraging results (46, 47). Another HIF-1α inhibitor topotecan is currently being tested in clinical trials for late-stage solid tumors. After 1 week of treatment, DCE-MRI imaging demonstrated a reduction in tumor blood flow and permeability, indicating effective suppression of HIF-1α expression in late-stage solid tumors (48). The CAIX inhibitor SLC-0111 has shown a good safety profile in phase I trials for treatment-experienced patients with late-stage solid tumors, even at high doses of 1,000 mg per day. Some patients treated with SLC-0111 have exhibited prolonged stable disease (SD). In addition, SLC-0111 augmented the effects of immune checkpoint blockade in preclinical models of melanoma and breast cancer. Nevertheless, further clinical studies are warranted to explore the efficacy and safety of SLC-0111 in a larger patient population (49).

To summarize, inhibiting the HIF-1 and CAIX pathways in HNSCC and other solid tumors can disrupt the adaptive mechanisms of tumor cells to hypoxia, overcome treatment resistance, and augment the efficacy of existing therapies. However, it is crucial to fully elucidate the complex mechanisms underlying tumor hypoxia and develop effective and safe targeted therapies for HNSCC patients. In addition, combination of hypoxia-targeting agents with other treatment modalities, such as radiation and chemotherapy, should be explored to optimize treatment outcomes and improve patient survival.

### 2.3. Targeting tumor-promoting chronic inflammation

Inflammation is a dynamic defense mechanism that occurs in response to harmful stimuli, and involves biological, chemical, and physical factors. The primary objective of the inflammatory response is to eliminate damage and facilitate tissue regeneration (50). However, inflammation can also contribute to the progression of certain diseases by exacerbating tissue damage. Chronic inflammation in particular is characterized by prolonged cycles of tissue destruction and regeneration (51). Furthermore, inflammation plays a significant role in tumor development and progression, and tumor cells in turn can enhance inflammatory responses (52). The microenvironment of HNSCC is rich in inflammatory mediators that may promote tumorigenesis, and are therefore ideal targets for innovative cancer therapies.

#### 2.3.1. Targeting the COX-2 pathway

Cyclooxygenase (COX) enzyme exists as COX-1 and COX-2 isoforms. It has the function of converting arachidonic acid into prostaglandins (PG) (53). COX-1 is constitutively expressed in most cells and is involved in physiological functions such as platelet aggregation. On the other hand, COX-2 is an inducible enzyme that is upregulated only in response to inflammation and other pathological stimuli. In addition, COX-2 is aberrantly expressed in pre-cancerous and cancerous lesions, and its overexpression can promote carcinogenesis (54). The oncogenic effect of COX-2 is primarily mediated through the release of the pro-inflammatory mediator PGE2 (55). Numerous studies have demonstrated the significant role of the COX-2/PGE2 pathway in the progression of HNSCC. High expression levels of COX-2 and PGE2 in HNSCC have been associated with worse prognosis, lymph node involvement, advanced histological grade, local tumor recurrence, and lower survival rate (56). COX-2 and PGE2 enhance migration of OSCC cells by upregulating intercellular adhesion molecule-1 (ICAM-1), a surface glycoprotein involved in cell-to-cell adhesion (57, 58). In addition, both COX-2 and PGE2 regulate tumor angiogenesis by modulating VEGF or directly influencing endothelial cell proliferation (59). COX-2 expression is also correlated with lymph node metastasis and disease progression in nasopharyngeal carcinoma (NPC), and co-expression of COX-2/VEGF-C in OSCC has been associated with the generation of lymphatic vessels (60, 61). Furthermore, PGE2 promotes the maturation of regulatory T cells (Tregs) and facilitates the recruitment of MDSCs to the tumor tissues, which suppresses the anti-tumor immune response and promotes tumor growth (62).

The above findings suggest that COX-2 is a promising therapeutic target in cancer (63). Indeed, the COX-2 inhibitors tested so far have demonstrated high treatment efficacy with acceptable side effects compared to traditional anti-cancer therapies (64). In addition, COX-2 inhibitors can also increase tumor sensitivity to radiation and chemotherapy. Due to the simultaneous inhibition of COX-1 and COX-2, non-selective NSAIDs not only fail to achieve the anti-inflammatory and analgesic purpose, but also cause serious adverse effects, such as gastrointestinal tract damage and platelet dysfunction. On the other hand, selective NSAIDs only inhibit COX-2 and does not affect the protective effects of COX-1-catalyzed prostaglandins on the gastrointestinal tract and platelets, thus greatly reducing the risk of gastrointestinal side effects (65). However, NSAIDs that selectively target COX-2, including celecoxib and rofecoxib, cause minimal damage to the GI, and have been widely tested in clinical trials (66). For instance, rofecoxib has been shown to reduce neo-angiogenesis in colorectal cancer patients with liver metastasis (67). In a phase II clinical trial evaluating the efficacy and safety of celecoxib in advanced cancer patients with cachexia, the body weight and tumor necrosis factor (TNF-α) levels improved following celecoxib treatment. These findings suggest that celecoxib could be an effective monotherapy for cancer-related cachexia (68).

#### 2.3.2. Targeting tumor necrosis factor alpha

Tumor necrosis factor alpha (TNF-α) is a cytokine that plays a critical role in regulating inflammation, immunity, cellular homeostasis, and tumor progression (69). Recent studies show that TNF-α is one of the key mediators of cancer-related inflammation and acts as a tumor-promoting factor (70). It exerts its effects through TNF receptor 2 (TNFR-2) and TNF receptor 1 (TNFR-1). While TNFR-2 has higher affinity, it is mainly expressed on immune cells. On the other hand, TNFR-1 is expressed ubiquitously and initiates most of the biological activities of TNF-α (71). In addition, TNFR-1 is a dual-action receptor that relays both apoptotic and survival signals, and TNFR-1 activation also contributes to pro-inflammatory responses (72). Overexpression of TNF-α in HNSCC is associated with higher proliferative potency (73), and inhibiting TNF-α in oral cancer suppressed tumor growth. Furthermore, TNF-α is a mediator of pain perception and inflammation in oral cancer, and TNF-α blockade can potentially alleviate oral cancer-related pain (74, 75). TNF-α promotes HNSCC progression by upregulating MMP-9, which in turn enhances tumor migration and invasion by facilitating TGF-β1-induced EMT (76, 77). Moreover, TNF-α also increases the metastatic potential of HNSCC cells by upregulating CCR6 and CXCR-4 (78, 79).

Tumor necrosis factor alpha inhibitors, such as infliximab and etanercept, have been widely evaluated in clinical trials for various cancers and have shown promising results. In a phase II trial, infliximab showed therapeutic effects in renal cell carcinoma (RCC) patients. Infliximab may inhibit tumor cell proliferation by neutralizing TNF-α or inducing TNF-α-dependent apoptosis by depriving cells of the cytokine. Lower circulating levels of TNF-α can stabilize tumor growth (80). In another clinical study evaluating tolerance and biological effects in advanced cancer patients, infliximab was found to be safe and well-tolerated without dose-limiting toxic reactions. Etanercept has also demonstrated therapeutic efficacy and safety in phase II studies on recurrent ovarian cancer and metastatic breast cancer (81).

### 2.4. Targeting the tumor immune system

Immune cells are an important component of the TME, and exert both anti-tumorigenic and pro-tumorigenic effects. The MDSCs and TAMs are immunosuppressive cells that promote tumor growth and aid in immune evasion. The role of these cell types in HNSCC and their therapeutic potential have been discussed in greater detail below.

#### 2.4.1. Targeting tumor-associated macrophages

Most TAMs originate from the bone marrow and infiltrate into the tumor via peripheral blood (82). Macrophages can be classified into the classically activated M1 macrophages and alternatively activated M2 macrophages. M1 macrophages exhibit enhanced antigen presentation and lysosomal activity, and promote Th1 responses. They also secrete chemokines (e.g., TNF-α, iNOS) involved in immune activation and phagocytosis to counteract tumor growth. TAMs predominantly display the M2 phenotype, and produce pro-oncogenic factors (IL-10, IL-4, TGF-β, VEGF, and MMP) that drive tumor growth, metastasis, angiogenesis, and immune evasion (83, 84).

Haque et al. (85) found that CD206+ TAMs promote proliferation of oral tumor cells through EGF signaling. In addition, TAMs also play a role in regulating the adhesion, migration, and invasion of HNSCC cells, promote metastasis by supporting the generation of blood vessels and lymphatic vessels, and contribute to tumor progression via immunosuppression (86). In laryngeal squamous cell carcinoma, M2 macrophages activate JAK/STAT signaling to produce IL-10, which upregulates the immune checkpoint PD-L1. TAMs can directly inhibit T cell activation and proliferation, and induce T cell apoptosis via PD-L1. Immune checkpoint blockade through PD-1/PD-L1 inhibitors has been highly effective in various cancers (87).

Head and neck squamous cell carcinoma cells and TAMs have a mutually synergistic relationship. While the tumor cells release CCL2 to recruit monocytes and induce their differentiation and polarization to M2 macrophages, the latter release epidermal growth factor (EGF) that upregulates CCL2 expression in tumor cells (88). Numerous TAM-targeting drugs have been developed that are currently in the clinical phase of testing. CCL2/CCR2 inhibitors, such as carlumab (CNTO 888) (89) and PF-04136309 (90), modulate macrophage recruitment and differentiation, and have been tested in clinical trials. CSF-1 receptor (CSF-1R), a transmembrane tyrosine kinase receptor, plays a crucial role in regulating TAM development, morphology, survival, and function after binding with CSF-1 (91). Several CSF-1 and CSF-1R inhibitors, such as emactuzumab (RG-7155) (92), AMG-820 (93), and pexidartinib (PLX3397) (94), are currently undergoing clinical trials. In addition, reprogramming TAMs from the pro-tumor M2 phenotype to the anti-tumor M1 phenotype is a promising therapeutic strategy. RRx-001 is an SIRP-a and CD47 inhibitor that can repolarize TAMs to the M1 phenotype, and clinical trials conducted so far on cancer patients have been encouraging (95). Inhibitors of the CCL2/CCR2 axis and CSF-1/CSF-1R signaling also modulate macrophage recruitment and differentiation, and have shown promising results in preclinical and clinical studies. Therefore, elucidating the complex interactions between TAMs and the TME will help in the development of effective therapies for HNSCC and other cancers (Table 1).

**Table 1:** TAMs targeting therapies.

Target site	Substance	Cancer type	Mechanism of action	Phase
CCL2/CCR2 axis	Carlumab (CNTO 888)	Prostate cancer	Suppress the expression of CCL2	II
CCL2/CCR2 axis	PF-04136309	Pancreatic cancer	Blockade of CCR2	Ib
CSF-1/CSF-1R	Emactuzumab	Solid tumors	Blockade of CSF-1R	I
CSF-1/CSF-1R	AMG-820	Solid tumors	Blockade of CSF-1R	I
CSF-1/CSF-1R	Pexidartinib	Tenosynovial giant cell tumor	Blockade of CSF-1R	III
CD47 and SIRP-a	Bromonitrozidine (RRx-001)	Colorectal cancer	Macrophage repolarizing	II
NA	Zoledronic acid	Breast cancer	Depletion of M2-like TAMs	III

#### 2.4.2. Targeting myeloid-derived suppressor cells

Myeloid-derived suppressor cells constitute a heterogeneous population of cells that morphologically resemble immature granulocytes, monocytes, and dendritic cells (DCs) (96). The MDSCs are normally scarce but their numbers increase significantly during early or advanced stages of cancer (97). MDSCs are primarily recruited to the TME through the CXCR2 ligand, which is overexpressed in various cancers (98). They inhibit T cell-mediated immunity through multiple mechanisms. For instance, MDSCs interfere with the supply of amino acids (such as L-arginine and L-citrulline), which is necessary for T cell proliferation and activation. In addition, the MDSCs produce high levels of reactive oxygen species (ROS), which interact with nitric oxide (NO) to generate peroxynitrite (ONOO−) radicals that inhibit T cell activation and proliferation (99). Moreover, MDSCs promote tumor angiogenesis by expressing VEGF, and the latter recruits MDSCs through the VEGF receptor (VEGFR) expressed on the cells’ surface (100).

The accumulation of MDSCs in the tumor tissues is closely associated with clinical outcomes and generally indicates poor prognosis. MDSCs are abundant in HNSCC tissues (101), and promote tumor progression in HNSCC through various mechanisms, including proliferation, apoptosis resistance, migration, invasion, EMT, and vasculogenic mimicry formation (VM). Tumor cells also induce immunosuppression by upregulating arginase 1 (ARG1) and inducible nitric oxide synthase (iNOS) in the MDSCs (102). Detection of circulating MDSCs in patients with thyroid nodules using flow cytometry is a novel approach for the evaluating cancer risk and severity, and may even serve as a useful tool for predicting the tumor stage and recurrence risk of HNSCC (103). Fugle et al. (104) demonstrated that functional inhibition of MDSCs in mice delayed the onset of oral cancer. Signal transducer and activator of transcription 1 (STAT1) is a transcription factor involved in a wide variety of immunological responses (104). Ryan et al. (105) showed that inhibiting accumulation of MDSCs in HNSCC through STAT1 promotion facilitated T cell-mediated anti-tumor immune response.

Current treatment strategies targeting MDSCs mainly focus on (1) depletion of MDSCs, (2) inducing differentiation and maturation of MDSCs, and (3) inhibition of the immunosuppressive functions of MDSCs. For instance, most colorectal cancer patients showed a decrease in MDSC numbers after first-line combination therapy with 5-fluorouracil, oxaliplatin, and bevacizumab (FOLFOX-bevacizumab), which was associated with improved survival outcomes (106). Furthermore, all-trans retinoic acid (ATRA) can induceefore decrease their numbers in circulation. The combination of ipilimumab and ATRA significantly reduced the number of circulating MDSCs in melanoma patients compared to ipilimumab monotherapy (107). Nrf2 plays a crucial role in regulating the expression of antioxidant enzymes and protects cells against free radical damage. The synthetic triterpenoid compound CCDO-Me reduced the production of ROS by MDSCs through Nrf2 upregulation, and reversed their immunosuppressive effects (108, 109). Several drugs that target the above aspects of MDSCs are currently in clinical trials. Furthermore, phosphodiesterase-5 (PDE5) inhibitors such as sildenafil, tadalafil, and vardenafil can reduce the levels of ARG1 and iNOS, thereby reversing MDSC-mediated immune suppression, reducing inflammation in the TME, and reactivating anti-tumor T cells and NK cells (110–112). In one clinical trial, tadalafil significantly reduced the number of intra-tumoral and circulating MDSCs and Tregs in HNSCC patients, and was well-tolerated. Chemotherapeutic agents can also effectively deplete MDSCs.

### 2.5. Targeting tumor angiogenesis

The rapid proliferation of tumor cells is accompanied by generation of new blood vessels that supply adequate nutrients, oxygen, and growth factors for sustaining tumor growth and facilitating dissemination of tumor cells (113, 114). Neo-angiogenesis involves tumor endothelial cells (TECs) and surrounding perivascular cells. TECs exhibit genetic abnormalities and are resistant to anti-angiogenic drugs (115). Naito et al. (116) showed that the recalcitrance of TECs to antiangiogenic drugs may contribute to tumor resistance. In addition, endothelial cells play a significant role in tumor progression and metastasis. The hypoxic conditions in the tumor tissue induce the production of VEGF, which initiates tumor angiogenesis and confers resistance to hypoxia. VEGF exerts its effect upon binding to its receptors (VEGFR-1, VEGFR-2, and VEGFR-3). VEGFR-1 and VEGFR-2 are expressed in the blood vessels, while VEGFR-3 is expressed in the lymphatic endothelium (117). Elevated VEGF expression in HNSCC has diagnostic and prognostic value. VEGF activates the VEGF receptors on the surface of the neighboring endothelial cells through paracrine signaling, which stimulates their migration and proliferation, and induces angiogenesis (118). During neovascular expansion, endothelial cells expressing high levels of VEGFR become tip cells and promote angiogenesis by interacting with delta-like ligand 4 (DLL4) and angiopoietin 2 (ANGPT2) (119). Sun et al. (120) demonstrated that inhibition of VEGF/VEGFR2 signaling with the flavonoid B2PB2 suppressed angiogenesis and growth in the OSCC cell line SCC-25. It also decreased the viability, invasion, migration, and EMT of the tumor cells, and promoted apoptosis (120). Under normal circumstances, endothelial cells remain quiescent and proliferate once every 150 days. However, increased expression of VEGF in response to various pathological stimuli can induce endothelial cell-mediated angiogenesis. Chen et al. and Wu et al. have shown that inhibiting VEGF expression can suppress migration and angiogenesis in NPC cells (121, 122). Anti-angiogenic drugs targeting VEGF/VEGFR, including bevacizumab (123), apatinib (124), vandetanib (125), AMG 706 (126), pazopanib (127), axitinib (128), famitinib (129), lenvatinib (130), cabozantinib (131), and regorafenib (132). Most of these drugs have shown therapeutic effects against various cancers, and could be considered for HNSCC treatment. There are others that need to be further explored because of toxicity or efficacy (Table 2).

**Table 2:** Antiangiogenic agents targeting vascular endothelial growth factor signaling in clinical trials.

Regimen	Phase	Sample	Cancer typle	Outcome
Bevacizumab, carboplatin and paclitaxel	IV	398	Ovarian cancer	Median PFS: 20.8 months median OS: 41.1 months
Apatinib vs. Placebo	III	92	Thyroid cancer	Median PFS: 22.2 months ORR: 54.3% DCR: 58.7%
Vandetanib and everolimus	I	80	Solid tumors	Median PFS: 4.1 months median OS: 10.5 months
Motesanib，paclitaxel, and carboplatin	III	401	Nonsquamous non-small-cell lung cancer	Median PFS: 5.6 months ORR: 60.1%
Pazopanib	II	168	Thyroid carcinoma	Best response rate: 35.6% DCR: 89.4%
Axitinib and pembrolizumab	Ib	52	Renal-cell carcinoma	Median PFS: 23.5 months ORR: 73.1%
Famitinib and camrelizumab	II	33	Cervical squamous cell carcinoma	Median PFS: 10.3 months 12-month duration of response rate: 74.1%
Lenvatinib	II	52	Thyroid cancer	1 year overall survival rate: 11.9% ORR: 11.9% DCR: 73.8%
Cabozantinib	III	258	Thyroid cancer	Median PFS: 11.0 months
Regorafenib	II	39	Biliary tract cancer	ORR: 9.1% DCR: 63.6%

PFS, progression-free survival; OS, overall survival; ORR, objective response rate; DCR, disease control rate.

### 2.6. Targeting other factors in the TME

In addition to the above TME components, there are many other TME components (e.g., cancer stem cells, microorganism, and mechanical microenvironment) that have received less attention but may also be therapeutic targets for tumors. Cancer Stem Cells (CSCs) constitute a small portion of malignant cells and serve as tumor-initiation cells, propelling tumor development (133). CSCs possess a range of functions, including plasticity, quiescence, and self-renewal, enabling them to regulate tumor growth, metastasis, survival, recurrence, and resistance to cancer treatment through specific signaling pathways (134, 135). Specific molecules have been identified as markers for CSCs in HNSCC, such as Aldehyde Dehydrogenase (ALDH) and CD44. ALDH+ CD44+ cancer cells are considered CSCs in HNSCC and exhibit increased tumorigenicity through the aberrant activation of the PI3K/mTOR signaling pathway and upregulation of SOX2 expression (136). The tumor microenvironment (TME) harbors microorganisms, and the microbial communities that influence tumor progression and are associated with tumors are referred to as the tumor microbiota (137). Tumors can create more suitable conditions for microbial survival and remodeling of microbial profiles, while microbes can also contribute to tumorigenesis and progression by establishing an inflammatory milieu and influencing host immunity, and unlike normal tissues where the balance of the microbiota contributes to the defense against tissue pathology, the microbiota in the TME affects tumor progression and therapy (138). The mechanical microenvironment is also part of the TME.

## 3. Conclusion

In this review, we have summarized the role of hypoxia, inflammatory response, immune cells, and angiogenesis in the progression of HNSCC, and discussed novel therapeutic strategies targeting these components. In recent decades, the focus of cancer treatment has steadily shifted to the TME, and numerous clinical trials are currently underway to validate the efficacy and safety of anti-cancer agents targeting the cells and factors that comprise the TME. Several of these targeted therapies have demonstrated promising clinical outcomes. However, disrupting the interactions between tumor cells and the TME often yield suboptimal results. It has been realized that TME is a complex ecosystem, full of heterogeneity, that can affect almost every aspect of cancer biology. At the same time, the advantages of targeted drugs over conventional drugs have been deeply understood, and the efficacy of many drugs targeting the TME in tumors has brought home the potential of the TME for tumor therapy. Therefore, there is an urgent need to elucidate the relationship between HNSCC and TME in more detail, with a focus on targeting the key components that promote tumor growth within the TME, to find more targets for treating tumors, to improve and refine the drugs in current clinical trials, and to develop more effective antitumor strategies.

## Author contributions

ZG: Conceptualization, Investigation, Writing – original draft. KL: Conceptualization, Investigation, Writing – original draft. PL: Conceptualization, Investigation, Writing – original draft. XZ: Writing – original draft. JL: Project administration, Supervision, Writing – review & editing. XZ: Funding acquisition, Project administration, Supervision, Writing – review & editing. PZ: Funding acquisition, Project administration, Supervision, Writing – review & editing.

## Funding

The author(s) declare financial support was received for the research, authorship, and/or publication of this article.

The present study was supported in part by Guangdong Basic and Applied Basic Research Foundation (2021A1515010970); Shenzhen Innovation of Science and Technology Commission (No. JCYJ20210324132407019, LGKCYLWS2022002, LGWJ2021-118, LGKCYLWS2021000027); and Shenzhen Key Medical Discipline Construction Fund (No. SZXK039).

## Conflict of interest

The authors declare that the research was conducted in the absence of any commercial or financial relationships that could be construed as a potential conflict of interest.

## Publisher’s note

All claims expressed in this article are solely those of the authors and do not necessarily represent those of their affiliated organizations, or those of the publisher, the editors and the reviewers. Any product that may be evaluated in this article, or claim that may be made by its manufacturer, is not guaranteed or endorsed by the publisher.
